# Adult Health Status Among Native American Families Participating in the Growing Resilience Home Garden Study

**DOI:** 10.5888/pcd16.190021

**Published:** 2019-08-22

**Authors:** Christine M. Porter, Alyssa M. Wechsler, Shawn J. Hime, Felix Naschold

**Affiliations:** 1 Division of Kinesiology and Health, University of Wyoming, Laramie, Wyoming; 2 Wyoming Survey & Analysis Center, University of Wyoming, Laramie, Wyoming; 3 Department of Economics, University of Wyoming, Laramie, Wyoming

## Abstract

Northern Arapaho and Eastern Shoshone tribes sharing the Wind River Indian Reservation (WRIR) in Wyoming reportedly die 30 years earlier than whites in the state. We analyzed data on the health status of 176 adults from 96 families who participated in a randomized controlled trial to assess health effects of home gardens. Measures of body mass index, waist circumference, blood pressure, hemoglobin A1c, vitamin D, low-density lipoprotein cholesterol, and household food security were collected from participating adults before the intervention. Results indicated that this group has considerably worse health status than average US adults and also fares worse than average American Indians/Alaska Natives. To help improve these disparities, Native Americans need access to appropriate and effective means of health promotion.

**Summary**

**What is already known on this topic?**

As survivors of historical trauma, the Northern Arapaho and Eastern Shoshone tribes sharing the Wind River Indian Reservation (WRIR) in Wyoming have significant health disparities, but very little data on this population have been published.

**What is added by this report?**

This research is the first to share data on adult health status in the WRIR. We found that this group of adults had worse average health status than adults at state and national levels, including American Indians/Alaska Natives as a whole.

**What are the implications for public health practice?**

This nation’s traumatic colonization history suggests that health promotion interventions alone cannot reduce health disparities this population. Still, access to effective interventions should be available to every Native family.

## Objective

As survivors of historical trauma, the Northern Arapaho and Eastern Shoshone tribes sharing the WRIR in Wyoming reportedly die 30 years earlier than whites in the state (1). However, only 2 peer-reviewed studies with WRIR health data have been published, both of which focused on children (2,3). This research is the first to share data on adult health status in the WRIR. We analyzed biometric, whole-blood, and survey-based data that were collected on 176 adults from 96 families who enrolled in Growing Resilience, a randomized controlled trial to assess the effects of home food gardens on health.

## Methods

Families were eligible to enroll in Growing Resilience if they 1) lived in the WRIR, 2) had at least 1 participating family member identifying as enrolled in a federally recognized tribe, 3) had at least 2 adults (or 1 for single-adult families) willing to participate in 2 years of gardening and health data collection, and 4) were interested in receiving and maintaining a home food garden but had not had a food garden over 30 square feet the previous year. Project partners aimed to recruit 100 families over 3 years, 2016–2018, and administered screening forms for eligibility. Recruitment and promotion activities included newspaper advertisements, flyers, open houses, and word of mouth. Overall, 119 interested families were eligible and were invited to enroll in the study. Of those, 96 families (81%) enrolled in the study immediately before their first health data collection. We report results for the 176 adults (aged ≥20 y) in those families.

We collected biometric, blood draw, and survey (up to 46 questions) data. Researchers measured height and weight for body mass index calculations using a Seca 213 Mobile Stadiometer (Seca) and a Tanita SC-331S Body Composition Analyzer (Tanita); assessed blood pressure using an Omron 10 Plus Series upper arm blood pressure monitor with ComFit Cuff (Omron Healthcare); and measured waist circumference using a Gulick II tape measure (Gulick). Whole blood analysis included hemoglobin A1c (HbA1c), vitamin D, and low-density lipoprotein (LDL) cholesterol (conducted by LabCorp). We tested for sex differences in all health indicators using a 2-sample Kolmogorov–Smirnov test for equality of distributions. The survey included demographic questions, and heads of households were administered the US Department of Agriculture household food security 6-question module (adapted to report on previous month) (4). Study protocol details are available elsewhere (5). The Growing Resilience study was approved by the University of Wyoming institutional review board. 

## Results

Of the 176 adults, 63% were female and 37% were male. Participant ages were evenly distributed (19%–25% per category) with the exception of adults aged 60 to 69 years (12%) and those aged 70 or older (<3%). Of the 170 adults who identified their tribal affiliation, 44% identified as Northern Arapaho, 40% as Eastern Shoshone, 3% as both, and 13% as another tribe. Most adults (91%) were overweight or obese, and more than one-third (37%) were categorized as obese class II or III (Table). Just over three-quarters had high blood pressure, more than half of whom had stage 2 hypertension.

**Table:** Outcomes From Biometric, Whole Blood, and Survey Measures, Participants of the Growing Resilience Project, Wind River Indian Reservation, Wyoming, 2016–2018

Measure	Female	Male	Overall
	%		
**Body mass index (kg/m^2^), n = 174 adults (110 female, 64 male)**			
Underweight (<18.5)	1.8	0	1.1
Normal weight (18.5 to <25)	6.4	9.4	7.5
Overweight (25 to <30)	24.6	31.3	27.0
Obese class I (30 to <35)	26.4	29.7	27.6
Obese class II (35 to <40)	23.6	15.6	20.7
Obese class III (≥40)	17.3	14.1	16.1
**BP, n = 176 adults (111 female, 65 male)^a^**			
Normal BP (systolic BP <120 mm Hg AND diastolic BP <80 mm Hg)	19.8	12.3	17.0
Prehypertension (systolic BP 120–129 mm Hg AND diastolic BP <80 mm Hg)	9.0	4.6	7.4
Stage 1 hypertension (systolic BP 130–139 mm Hg OR diastolic BP 80–89 mm Hg)	36.9	21.6	31.3
Stage 2 hypertension (systolic BP ≥140 mm Hg OR diastolic BP ≥90 mm Hg)	34.2	61.6	44.3
**Diabetes status, n = 167 adults (105 female, 62 male)**			
Normal hemoglobin A1c (<5.7%)	51.4	43.6	48.5
Prediabetes (hemoglobin A1c 5.7%–6.4%)	31.4	35.5	32.9
Controlled diabetes (self-identifies as having diabetes AND has hemoglobin A1c <7%)	5.7	9.7	7.2
Not controlled diabetes (self-identifies as having diabetes AND has hemoglobin A1c ≥7%)	8.6	9.7	9.0
Undiagnosed diabetes (does not self-identify as having diabetes AND has hemoglobin A1c ≥6.5%)	2.9	1.6	2.4
**Vitamin D, n = 167 adults (105 female, 62 male)**			
Adequate vitamin D (≥30 ng/mL)	8.6	1.6	6.0
Possibly insufficient vitamin D (>20 to <30 ng/mL)	13.3	17.7	15.0
Vitamin D deficient (≤20 ng/mL)	78.1	80.7	79.0
**LDL Cholesterol, n = 163 (104 female, 59 male)**			
Normal LDL cholesterol (<100 mg/dL)	47.1	57.6	50.9
High LDL cholesterol (≥100 mg/dL)	52.9	42.4	49.1
**Waist circumference, n = 176 adults (111 female, 65 male)**			
Healthy waist circumference among women (<35 in) and men (<40 in)	5.4	20.0	NA
**Household food security previous month, n = 94 households**			
High food security (raw score of 0 affirmatives)	NA	NA	19.1
Marginal food security (1 affirmative)	NA	NA	16.0
Low food security (2–4 affirmatives)	NA	NA	43.6
Very low food security (5 affirmatives; 6th question was not asked)	NA	NA	21.3

Abbreviations: BP, blood pressure; LDL, low-density lipoprotein; NA, not applicable.

a Using 2-sample Kolmogorov–Smirnov tests for equality of distributions to test for sex differences in the individual health measures in this tables showed only BP as significantly different, with men having higher systolic (D = 0.368, *P* < .001) and diastolic (D = 0.278, *P* = .002) BP.

Blood measures indicated that a little more than half of adults for whom LDL cholesterol results were collected (n = 163) had normal levels, and nearly 80% of adults had deficient vitamin D levels (≤20 ng/mL; n = 167). Almost half of adults had normal HbA1c levels, one-third had levels that indicated prediabetes, and 19% had diabetes (n = 167). Of those with diabetes, 4 were undiagnosed (ie, did not self-report as diabetic, but had HbA1c ≥6.5%; these participants were notified). Nearly all women (95%) and most men (80%) had waist circumference measures associated with higher risk of obesity-related disease.

The only measure in which a difference by sex was found was blood pressure; men had higher systolic (D = 0.368, *P* < .001) and diastolic (D = 0.278, *P* = .002) blood pressure than women. At the family level, 65% of the 94 responding heads of household reported being food insecure during the previous month. Of those, 20 households (21%) had very low food security, and 15 households (16%) had marginal food security.

## Discussion

These members of the Northern Arapaho and Eastern Shoshone living on the WRIR in Wyoming suffer worse average health status than adults at state and national levels, including among American Indians/Alaska Natives (AIs/ANs) at large (Figure). Their obesity rate is 70% higher than the national AI/AN average (6), hypertension rates are more than double (7), and high LDL cholesterol rates are one-third higher (8). The diabetes rate is 2.3 times the Wyoming average and 23% higher than AI/AN averages (6). Their 65% food insecurity rate is more than 10 times the national 30-day rate (9).

**Figure:**
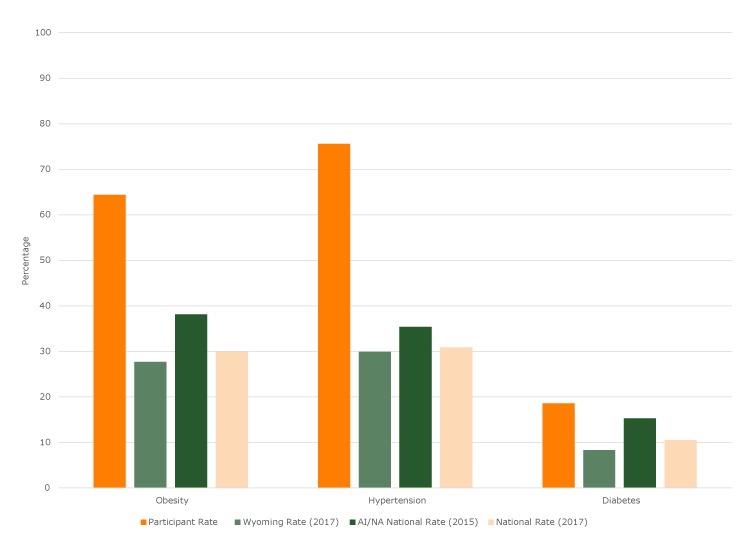
Rates of obesity, hypertension, and diabetes among adult Growing Resilience participants before any intervention compared with state and national rates. State and national data are from the Behavioral Risk Factor Surveillance System (6,7). Abbreviation: AI/AN, American Indian/Alaska Native.

**Table:** 

Metric	Study Participant Rates, 2016–2018	Rates in Wyoming, 2017	American Indian/Alaska Native National Rates, 2015	US National Rates, 2017
	%			
Obesity	64	28	38	30
Hypertension	76	30	35	31
Diabetes	19	8	15	11

Our study has limitations. The small sample size constrains analyses by sex or age, and data were not taken from a random sample. WRIR-wide estimates reported by tribal health affiliates (10) and findings in this sample did roughly align for obesity (71% reported vs 64.4% here) and diagnosed diabetes (12% reported vs 16.2% here). Although our results may not be fully generalizable to the WRIR adult population, the severity of poor health in this sample suggests that WRIR communities are living with enormous health challenges, many of which can result in death if left untreated (11). These data also currently comprise the most complete set that is publicly available about adult health status in WRIR.

To begin addressing these challenges and reducing these health disparities, Native American families need access to multiple and effective means to control and enhance health (12,13). The Growing Resilience study is assessing to what extent home gardening provides one such means. Although this nation’s traumatic colonization history suggests health promotion interventions alone cannot remedy such large health disparities, access to effective interventions should be available to every Native family.
